# Frontline, Essential, and Invisible: The Needs of Low-Wage Workers in Hospital Settings During COVID-19

**DOI:** 10.1177/21650799221108490

**Published:** 2022-07-17

**Authors:** Lisa de Saxe Zerden, Erica L. Richman, Brianna Lombardi, Alexandria B. Forte

**Affiliations:** 1 School of Social Work, The University of North Carolina at Chapel Hill; 2 Cecil G. Sheps Center for Health Services Research, The University of North Carolina at Chapel Hill; 3 Department of Family Medicine, Cecil G. Sheps Center for Health Services Research, The University of North Carolina at Chapel Hill

**Keywords:** behavioral/mental health/stress/anxiety/depression, COVID-19, health care worker/homecare worker, low-wage worker, qualitative research methods

## Abstract

**Background:**

Frontline health care workers are particularly vulnerable to burnout and diminished well-being as they endure COVID-19 pandemic-related stressors. While physicians and nurses are the public face of those experiencing burnout in hospitals, these stressors also affect low-wage workers such as food and housekeeping/janitorial service workers whose roles largely remain “invisible” when conceptualizing the essential health workforce and understanding their needs. This study sought to understand the experiences of frontline essential workers to better support them and prevent burnout.

**Methods:**

Using a semi-structured interview guide, we conducted 20 in-depth qualitative interviews with workers in three U.S. states. Thematic content analysis was conducted to code and analyze interviews.

**Results:**

Workers had an average of 5.8 years in their jobs, which included food services, housekeeping/janitorial, and patient transport roles. Analysis revealed four prominent stressors contributing to worker burnout: changes in duties and staff shortages, fear of contracting or transmitting COVID-19, desire for recognition of their job-related risk, and unclear communication on safety precautions and resources. Protective factors included paid time-off, mental health supports, sense of workplace pride, and self-coping strategies.

**Conclusion/Application to Practice:**

As health systems continue to grapple with care delivery in the context of COVID-19, identifying best practices to support *all* workers and prevent burnout is vital to the functioning and safety of hospitals. Further consideration is warranted to create policies and multipronged interventions to meet workers’ tangible needs while shifting the culture, so all members of the health workforce are seen and valued.

Estimates suggest the U.S. population has nearly 7 million underpaid but essential health care workers, including home health workers, medical and nursing assistants, food service workers, patient orderlies, housekeepers, patient sitters, and janitors (Kinder, 2020). These workers are more likely to be women, immigrants, and people of color (Kinder, 2020; Shulman, 2011). These types of workers are more likely to be in low-wage positions, experience heightened physical, mental, and financial hardships (Cubrich, 2020), and earn a national median hourly wage of US$13.48 (Kinder, 2020).

COVID-19 has caused significant burnout among health care providers and forced health systems to consider strategies to address added stress and burden (Dzau et al., 2020). Frontline health care workers are particularly vulnerable to burnout and diminished physical, emotional, and overall well-being as they regularly endure pandemic-related stressors that reverberate in their personal and professional lives (Chen et al., 2021). The impact of this stress has most often focused on those providing direct patient care (i.e., nurses and physicians) who are recognized as health care “heroes” (Bauchner & Easley, 2020; Stokes-Parish et al., 2020). However, equally heroic yet less lauded are workers who keep health systems clean, fed, and moving (Kinder, 2020). This invisibility is not just within the public’s conception of who makes up the health workforce, but also within the literature on burnout, worker satisfaction, well-being, and stress both in the United States and internationally where research remains scant (Chatti et al., 2019; Shukla et al., 2021).

Just as the pandemic has highlighted existing inequities affecting COVID-19-related morbidity and mortality (Khanijahani, 2021), disparities also exist in how frontline essential workers experience job-related stress in health settings (Cubrich, 2020). Furthermore, the economic hardships of COVID-19 are disproportionately experienced by those who are racial/ethnic minorities, women, younger, and have less education (Couch et al., 2020; Lee et al., 2021). Job precarity for those in service industries that require the worker to be physically present such as in food services were more likely to lose their jobs due to changes in workflow disruptions caused by COVID-19 (Couch et al., 2020; Tomer & Kane, 2020).

This study explored the unique experiences of low-wage essential, frontline workers across hospital settings to better understand the needs of these workers and identify strategies to support them during and beyond the pandemic. Frontlines hospital workers in housekeeping/janitorial services, food preparation or service, or other nonclinical functions were included. The purpose of this study was to identify the main sources of stress contributing to burnout among low-wage frontline workers in hospital systems during the COVID-19 pandemic as well as strategies health systems implemented to better support these workers.

## Methods

The study sample consisted of 20 low-wage workers recruited via snowball sampling methods from hospitals in California (*n* = 1), Colorado (*n* = 5), and North Carolina (*n* = 14). Guided by the Brookings Institute’s low-paid health jobs taxonomy (Ross & Bateman, 2019), this study focused on workers in food services, housekeeping/janitorial, and patient transport roles. Flyers were distributed via email to hospital employees known to the research team. Inclusionary criteria were based solely on job role/title and ability to participate in an interview in English; no one was excluded based on region, length of employment, or demographic characteristics. Participants were compensated with a US$50 e-gift card, and the study was approved by the institutional review board (IRB) at the University of North Carolina at Chapel Hill (IRB# 20-2601).

A semi-structured guide was used to facilitate interviews and was created with input from content experts and extant literature and consisted of 21 questions across four domains: (a) work and education, (b) COVID-19 resources provided by the employer, (c) homelife and job security, and (d) one open-ended question, “What do you want people to know about your work during the pandemic?” Interviews took place from February to June 2021. After participants consented, interviews were audio-recorded via Zoom and lasted approximately 20 minutes. Audio recordings were transcribed and checked for accuracy.

### Data Analysis

Thematic content analysis was conducted as all interviews were coded and analyzed by two research team members working independently following the six interactive phases of qualitative coding (Labra et al., 2019). These primary coders developed a codebook to generate initial codes based on contextual data. Codes were discussed iteratively by the full research team and collectively, coding discrepancies were reconciled.

## Results

The sample was on average 41.7 years old (*SD* = 13.2) and female (*n* = 12). Racial/ethnic demographic information was optional and thus includes missing data (*n =* 11). In terms of formal education, one respondent had some high school, 35% had a high school degree, 25% had some college or an associate’s degree, 30% had a college degree, and one participant had education beyond college. The type of employment varied but the sample represented housekeeping (*n* = 7) also known as environmental services, food services (*n* = 6), patient transport (*n* = 5), and front-desk roles (*n* = 2). On average, workers had been in their jobs for 5.8 years (range = 3 months–20 years); 80% of workers held their jobs before the pandemic and 20% were hired during the pandemic. The majority (70%) lived with others in their home, 15% lived alone, and the remainder did not disclose domestic arrangements. All participants indicated their basic needs were met (i.e., ability to pay for housing and utilities, did not run out of food in past month).

Overall, seven themes emerged, of which four involved stressors contributing to frontline workers’ sense of burnout during COVID-19 including (a) changes in duties and staff shortages, (b) fear of contracting or transmitting COVID-19, (c) desire for risks/importance of their jobs to be recognized, and (d) lack of clarity regarding COVID-19 resources/eligibility and perceived unequal distribution of resources. Three themes included factors participants identified as protective factors that buffered stressors, including the provision of paid time off for COVID-19-related circumstances, organizational efforts to provide mental health support, and self-coping strategies and pride in their jobs.

### Stressors

#### Theme 1: Changes in duties and staff shortages

All respondents described how COVID-19 changed their jobs. New positions were created (e.g., COVID-symptom screeners), elective procedures and appointments were paused, resources were pared down (e.g., cafeterias closed), and health systems were understaffed (Dzau et al., 2020). These changes generated additional strain on an already fatigued system (Chen et al., 2021). One respondent described the heightened demands in their job: “I had to work by myself upstairs and cook and do the cash register, there was a lot more work to do, and less people . . . definitely our workflow changed.” This theme was echoed by another respondent, who stated, “Yeah, we’re real short staffed . . . Definitely burnout. . . I feel like I’m doing maybe two or three-people’s job. Just saying, pretty much our job title meant nothing anymore.” Respondents described how these changes were not recognized by hospital administrators and why this lack of a understanding heightened their stress:

[W]e’re so short staffed, they expect me to do so much more than what I think I’m capable of doing. . . it’s not really a one-person job . . . then, if you don’t do it perfectly, then they have something to say “Oh, you know you’re not really doing your job blah, blah.” It’s a constant yammering about my job. It’s like “Dude, if I had another person working it wouldn’t be that bad!” [but] they’re not hiring anyone else.

#### Theme 2: Fear of COVID-19 for self and others

Contracting COVID-19 was a constant fear for participants’ personal safety as well as fear of transmitting the virus to others, leading to emotional exhaustion. Participants described their work environments as persistently stressful and carrying this stress home. One participant shared,

I have a minor child with a preexisting condition. I must be careful when I come home. It’s not just come on and hug on me, it’s completely different and makes me more careful so I don’t bring stuff home.

Physical and mental manifestations of work-related stress and exacerbated workers’ diminishing morale:

I felt the burnout. It got intense for a while, it was harder to help the people in the way you’d like to. . . It probably took a toll on everybody. . . sometimes [co-workers] have their families come have lunch with them and then just not being able to do that even didn’t help morale. . . It just doesn’t help your mentality, seeing it as soon as you get home, straight on the news again, after seeing it at work all day long.

Witnessing people who were severely ill or dying was emotionally draining:

I saw those nurses staying by the patients every day and working so hard to keep them alive. . . it would be a lot worse than it was for me. I had one patient, where I was able to go in and be a part of praying for that patient and she passed away within the next 24-48 hours. That impacted me.

#### Theme 3: Desire for recognition given demands and importance of their work

Participants felt their work in the hospital was underappreciated. Many wanted recognition about the roles they fill:

I wish [people] knew that it was stressful every day we wake up, we want to protect them, we want to protect ourselves. A lot of people are in a panic, and they probably take their stress out on hospital workers, but they need to understand that we’re in this together. When we ask you to pull your mask . . . it’s not to deny you of any rights or anything—it is to try to keep everyone safe.

The EVS crews and transport, food and nutrition—they’re all as important as the ER doctors, they’re all as important as all the nursing staff . . . without them, the nursing staff can’t do their job. . . it really takes the whole village and we’re just as essential, if not more.

Respondents did not feel their work was valued or seen as equally important as clinical staff:

A lot of the departments, they kind of look down on housekeeping because I don’t think they understand what we really do. [It] puts us in danger like everyone else in the hospital, sometimes even more. . .

I don’t think enough precaution is taken for the men and the women that work environments because they’re the ones actually doing the dirty work. They’re the ones cleaning those rooms, a deep clean when a patient is COVID positive, sometimes there [are] other duties. I don’t think they have the support they should . . . they have a dirty job, no pun intended.

#### Theme 4: Lack of clarity around resources and eligibility

Nearly every respondent identified stress stemming from unclear COVID-19 policies and perceived unequal distribution of resources. Respondents frequently mentioned hearing about resources that were available (or were previously) but were unsure of their eligibility. A shared sentiment was that higher status clinical employees had access to things that were not offered to service workers. For instance,

It’s made me fearful. I’m afraid that I’m going to catch the COVID. I don’t feel like they did a good job [discussing resources]. Well, I don’t know if it’s true or not, but I heard that they were paying the nurses and giving them COVID pay but they wasn’t giving it to the housekeepers.

One participant shared a perception of unequal distribution of hazard pay:

A lot of our nursing staff was for sure offered the hazard pay. And then everybody try to keep that on the downlow, though . . . we’re very big proponent of [what] it takes—a whole family and a whole house to run the hospital. So that’s not super awesome for the rest of us who are also just busting our butts just as hard.

The nurses, a lot of departments in the hospital got hazard pay and we in housekeeping we’ve never gotten it for some reason. I don’t know why, and I don’t feel that that’s fair . . . there’s nobody more exposed in that room that we are. I don’t feel that it’s fair that other departments get that hazard pay, and we’re not getting it.

Relatedly, one participant described a program for child care assistance but financial support for this program ended before they tried to claim this resource.

### Protective Factors to Buffer Stress and Burnout

Despite identified stressors, participants also articulated ways in which they felt supported during the pandemic.

#### Theme 5: Paid time off designated for COVID-related circumstances

Although health systems varied in the types of leave offered, the provision of paid time off (PTO) or flexible-leave options were tangible ways participants felt supported by their employers, which in turn, buffered stress related to COVID-19: for example, “I do know that if you test positive it doesn’t come out of your PTO; there’s a special fund for COVID to pay the employees, so it doesn’t affect PTO.” For many, personal circumstances made taking sick time without pay an infeasible option:

A lot of people just couldn’t afford to do that [take leave], they didn’t have any PTO so the hospital grants them a “negative” [day] . . . If you had zero and you had to take off two weeks, they would front you that PTO but you had to pay that back when you accrued it. In that sense, it’s not so much that you they gave money they just fronted you up to 160 hours, so that you didn’t go payless.

#### Theme 6: Organization-level supports for workers’ well-being and mental health

Other ways participants recognized their organization’s efforts was by addressing workers’ mental health needs. The most common type of organizational support was through communications to keep workers updated. Information was conveyed via various channels (e.g., e-mail, webinars, and verbally by managers). Organizational-level support was evident in the encouragement of mental health resources and offering workers opportunities for counseling.

#### Theme 7: Self-coping strategies and a sense of pride

In addition to organizational efforts, participants described how family and community supports protected against work stressors. One respondent said, “I’m a little better suited to get through it. Because I’ve got my family and it’s very important for me to work and try to remain positive.” Likewise, several participants conveyed an internal sense of pride given all they do to keep the hospital running:

We do everything we can to keep people safe that we clean every area . . . to keep us safe and the patients that go inside the hospital to be safe as well. And we are trying hard to maintain the environment and the hospital.

Beyond the responsibilities these workers have in keeping the hospital operational, respondents also described pride in their work because it directly impacts patients and care teams:

I have pride in what I do, and it doesn’t bother me one minute that people think like that [disparagingly about housekeeping]. The world is a sad place that people actually . . . look down on the person just because she’s a housekeeper. It’s a job and I have my pride, and nobody can make me feel bad about what I do. I’m proud of what I do, I make a difference.

You know, all the transporters, all the EVS workers, spend more time around patients, physically around patients than most doctors do. Doctors get a diagnosis and come in and save the day and you know, or even potentially not save the day. . . With the nursing staff, we’re in the room just as much as they are, and in that same space as the nurses, [we] are helping them out, we are getting them supplies, we’re moving their patients. . . We were right there.

One participant emphasized how his job gives him perspective:

To give an example, one day I was incredibly stressed out, I want to walk out of there and I went up to the front desk and there was [a patient] . . . she smiled at me and says, “Hey how ‘ya doing?” I’m thinking “Why am I complaining?” My job really levels me and grounds me that every time I get stressed out something like that happens, I realized there’s a lot more people in a lot worse off situation.

## Discussion

This study interviewed a vital group of health system employees regarding experiences of stress and burnout, as well as buffers and supports during COVID-19. Respondents described that being on the frontlines but not in direct care impacted their experiences in hospital settings. Many felt their work was not only underappreciated but also increasingly demanding with new duties, while short-staffed, and during a time of heightened personal safety risk. Participants found clear communication beneficial, especially regarding communication on resources, PTO, mental health supports, and self-coping strategies.

Prior research has well documented that the fear of contracting COVID-19 and the risk the virus poses to the individual, coworkers, and their family contributes to COVID-related stress (Larochelle, 2020; Nguyen et al., 2020). For example, a study of nurses found higher levels of COVID-19 fear were associated with decreased job satisfaction, increased psychological distress, and intentions to quit nursing (Labrague & de Los Santos, 2021). Although fear was not specifically measured among our sample, respondents’ fear of contracting COVID-19 and inadvertently transmitting the virus to others was a significant contributor to burnout. Health systems must consider these concerns not just for the benefit of workers’ mental health but also for how these thoughts contribute to workforce attrition and shortages in jobs essential to the operations and safety of care delivery.

Relatedly, respondents identified their sense of pride and belief in the intrinsic value of what they contribute to health systems as protective factors that sustained and motivated them to continue working during stressful circumstances. This type of positive validation is a quality that warrants more attention. Moreover, all members of the health system—clinical providers, administrators, and managers—need to demonstrate this appreciation for all levels of hospital workers, which in turn, can lead to improved worker outcomes. Research on organizational and positive psychology has shown the effects of positive validation to motivate employees to feel more engaged and committed to their organization (Meyers et al., 2013). The specific types of interventions that enhance employees’ well-being and can improve performance outcomes include acknowledgment of workers’ contributions and the implementation of human resource programs to support workers during COVID-19 such as clarified PTO policies, mental health supports, and other employee-assistance programs. Although health systems have deployed burnout-intervention programs during COVID-19 (e.g., Azizoddin et al., 2020; Zerden et al., 2022), specific attention to the needs of low-wage workers warrants nuanced consideration. Because the types of supports workers need vary, a multipronged approach is needed to address the different stressors workers face (i.e., financial, social, health-related, and/or psychological). Burnout prevention and intervention is not a one-size-fits-all approach; what works for some groups of workers might not work for all. Specifically, the supports put into place to address provider burnout might not resonate with those in housekeeping, food service, or related service jobs. Health systems need to consider how burnout prevention and intervention programs are designed, targeted, implemented, and evaluated. Health systems must consider how information is disseminated because not every type of worker accesses a computer regularly or relies on electronic information.

Given that previous literature has shown women and minorities are disproportionately represented in these types of low-wage jobs (Himmelstein & Venkataramani, 2019; Larochelle, 2020), we hypothesized these groups of workers would self-report higher rates of social needs. Although respondents in this study did not report financial, economic, housing, or food insecurity, this does not mean such needs were nonexistent. Participants might not have openly shared social needs due to social desirability in providing responses or related to the pride expressed for their work. However, some issues of the respondents had been neglected, including hazard pay and receiving additional compensation for expanded duties. Two ways to systemically address the financial needs of low-wage essential workers are first to identify them and their contributions (Tomer & Kane, 2020) and second, to investigate the commitment of health systems to provide livable wages and ensure economic security for all employees (Himmelstein & Venkataramani, 2019).

Although this study highlights the needs of frontline essential workers in food services, housekeeping, and patient transport, limitations must be considered. Findings are not generalizable given the sample size, types of workers, geographic locations within the United States, and no validated burnout measure. Generalizability is further limited based on hospital systems’ funding structures, labor regulations (e.g., unions), and COVID-19 response efforts; future work is necessary to validate these findings. Those who participated might differ from others who were unintentionally excluded given the physicality of these jobs requires workers to perform specific tasks away from computers and may have limited participation. Finally, the sample does not include those who were non-English speakers and reflects those who were part of the workforce 1 year into the pandemic. As such, our sample did not capture people who were laid off, left work electively, or were struggling in other ways. Future work should be done to elicit these perspectives.

**Applying Research to Practice Summary**

Little research has been conducted on the stressors and needs of workers in hospital service sectors such as housekeeping, food service, and patient transport roles despite their essential contributions to health service delivery and operations. During and beyond the COVID-19 pandemic, all frontline essential workers require concrete interventions to support their tangible needs (i.e., childcare and food) while also creating a shift in how these essential workers are recognized and valued across health systems. Workers in housekeeping, food service, and patient transport roles are integral to the functioning of the entire health system; without them, operations to clean, feed, and keep health systems moving would grind to a halt. These essential workers are indeed health care heroes who have persevered despite the strain, complexities, and disruptions COVID-19 has exacerbated. Given the U.S. health system relies on thousands of low-wage workers to keep hospitals functioning, identifying strategies to support this workforce and greater attention to their risk and protective factors for burnout and work-related stress warrants policy, workforce researchers, and health system administrators’ attention.
